# Relative Validity of an Online Herb and Spice Consumption Questionnaire

**DOI:** 10.3390/ijerph17082757

**Published:** 2020-04-16

**Authors:** Cynthia Blanton

**Affiliations:** Department of Nutrition and Dietetics, Idaho State University, Pocatello, ID 83201-8117, USA; blancynt@isu.edu; Tel.: +1-(208)-282-3937

**Keywords:** herbs, spices, questionnaire, validity

## Abstract

Culinary herbs and spices contribute bioactives to the diet, which act to reduce systemic inflammation and associated disease. Investigating the health effects of herb/spice consumption is hampered, however, by a scarcity of dietary assessment tools designed to collect herb/spice data. The objective of this study was to determine the relative validity of an online 28-item herb/spices intake questionnaire (HSQ). In randomized order, 62 volunteers residing in Idaho, USA, completed the online Diet History Questionnaire III + the HSQ followed one week later by one of two comparative methods: 7-day food records or three telephone-administered 24-h dietary recalls. Relative validity of the HSQ was tested two ways: (1) by comparing herb/spice intakes between the HSQ and comparator, and (2) by determining the correlation between herb/spice data and Healthy Eating Index 2015 score. The HSQ and both comparators identified black pepper, cinnamon and garlic powder as the three most commonly used herbs/spices. The HSQ captured significantly higher measures of the number and amount of herbs/spices consumed than the comparators. The number of herbs/spices consumed was significantly directly correlated with diet quality for the HSQ. These results support the ability of the HSQ to record general herb/spice use, yet suggest that further validation testing is needed.

## 1. Introduction

Consumption of herbs and spices is associated with improved health outcomes. Evidence supports the possible protective effects of culinary herbs and spices against oxidative damage, inflammation, cancer, infection and neurodegeneration [1,2,3,4]. Many of these protective effects are attributed to the high concentration of beneficial plant-derived compounds called polyphenols in herbs and spices [5]. For example, curcumin, a polyphenolic yellow pigment in turmeric, directly interacts with cell molecular targets to upregulate tumor-suppressor genes and inhibit inflammatory signaling pathways [6,7]. Cinnamon inhibits in vitro tumor cell proliferation and in vivo melanoma tumor growth [8].

Short-term human intervention trials demonstrate significant acute benefits of herb and spice intake [9,10,11,12,13]. Li et al. [10] demonstrated a significant reduction in plasma and urine malondialdehyde, a marker of oxidative damage, in volunteers fed hamburger containing ~11 g mixed spices vs. those fed a control hamburger. In a clinical trial testing the effect of oral curcumin on colorectal cancer in 44 men and women, a 4-g dose over 30 days resulted in a 40% reduction in the number of early neoplasms [9]. Lastly, a 2-mg acute dose of capsaicin vs. placebo was shown to increase resting energy expenditure, independent of measures of energy intake or circulating appetite hormones, in young obese men and women [13]. Beyond these and other short-term studies, few reports exist describing habitual intake of herbs and spices in relation to health outcomes. None of the large US national surveys or cohort studies (National Health and Nutrition Examination Survey, Nurses’ Health Study or Health Professionals Study) collect data on individual herb/spice intake.

Only one validation study of a tool designed to assess herb and spice intake is known to have been published. Carlsen et al. 2011, [14] validated herb and spice intakes from a paper 250-item food-frequency questionnaire against 28-d herb and spice records in a Norwegian population of middle-aged adults. Correlations between the methods were generally good for total intake and frequency of intake and portion size of commonly used herbs and spices.

Since the health benefits of spices and herbs likely result from long-term, consistent intake in small amounts [15], measuring their consumption requires a dietary assessment method that is detailed and precise. This study was designed as an initial validity assessment of a new online tool that captures measures of herb and spice consumption.

## 2. Materials and Methods

### 2.1. Study Design

The study sought to determine the relative validity of a new online herb and spice intake questionnaire (HSQ). A convenience sample of 62 adults completed, in randomized order, an online diet history questionnaire immediately followed by the HSQ and one of two comparative dietary assessment methods: 7-day online food records or three telephone-administered 24-h dietary recalls. The first group of participants was composed of 33 individuals who completed the study using food records between November 2018 and May 2019. The second group was composed of 29 individuals who completed the study using 24-h recalls between September and December 2019. Relative validity of the HSQ was evaluated in two ways: (1) by comparing herb and spice intake measurements between the HSQ and the comparative methods, and (2) by determining the relationship between herb/spice measurements and diet quality. The study protocol was approved by the Idaho State University Institutional Review Board, Human Subjects Committee (study number IRB-FY2019-80), and participants provided informed consent using an online form.

### 2.2. Participants

A convenience sample was recruited from the university and city communities of Pocatello, ID, USA, using university electronic bulletin boards, flyers and word-of-mouth. Pocatello is a one of the larger metropolitan areas in southeast Idaho, with a population of ~56,000 people of mostly white race (~85%) and a median household income of $44,000 USD in 2018. Approximately 90% of residents of Pocatello have graduated from high school and ~30% have a college diploma. Inclusion criteria were: age >18 years, owner of an email account and access to a computer with Internet. The exclusion criterion was self-reported adherence to a restricted diet (e.g., very low-kcal; exclusion of >2 food groups). This exclusion was intended to limit data collection to people following a typical diet. Interested persons contacted a research staff member to complete a telephone screening and eligible individuals were emailed a link to an online consent form. A research staff member determined the order of dietary assessment method (1st: Diet history questionnaire + HSQ, 2nd: Comparator method; or 1st: Comparator method, 2nd: Diet history questionnaire + HSQ) for each participant by coin flip. A research staff member emailed participants a link to their first dietary assessment activity and its completion was confirmed before a link to the second activity was emailed one week later. Participants were contacted to provide missing data or answer questions about their records as necessary. No personal data, such as demographic or health information, were collected from participants. Participant incentives were offered in a drawing for one of several gift cards at the end of the study.

### 2.3. Herb and Spice Questionnaire (HSQ)

The HSQ was developed using Qualtrics™ survey software (Provo, UT, USA) version 2019. Content validity was addressed by reviewing the literature pertaining to dietary surveys and herb and spice consumption reports in order to select the list of spices and herbs and the options for portion sizes and frequency of use [14,16,17]. Face validity was addressed by pilot testing the survey on faculty and staff and making modifications to the HSQ to improve the quality of information gathered. The survey queried consumption during the past month of 28 herbs and spices and included a fill-in option. The survey was created in the English language only.

The herbs and spices (vernacular and *scientific* names) included were:
AllspiceCelery seedCuminGingerOreganoTarragon*Pimenta dioica**Apium graveolens**Cuminum cyminum**Zingiber officinale**Origanum spp.**Artemisia dracunculus*AniseChili powderCurryMarjoramPaprikaThyme*Pimpinella anisum**Capsicum spp.**Murraya koenigii**Origanum majorana**Capsicum annuum**Thymus vulgaris*AnnattoCilantroDillMintParsleyTurmeric*Bixa orellana**Coriandrum sativum**Anethum graveolens**Mentha spp.**Petroselinum crispum**Curcuma longa*BasilCinnamonFennelMustardRosemary
*Ocimum basilicum**Cinnamomum spp.**Foeniculum vulgare**Brassica ssp.**Rosmarinus officinalis*Black pepperCloveGarlic PowderNutmegSage
*Piper nigrum**Syzygium aromaticum**Allium sativum**Myristica fragrans**Salvia officinalis**spp*: species.

A fill-in option was included.

Options for frequency of use were:

Never
Less than once per month
1, 2 or 3 times per month
1, 2, 3, 4, 5 or 6 times per week
1, 2 or >3 times per day

Options for portion size were:

<½ teaspoon; 1, 2 or >3 teaspoons
1, 2, 3, 4 or >5 shakes, with 3 options for shaker hole size (small, medium and large) and 2 options for container shape (rectangle, cylinder). Hole size and containers were depicted in photographs.
1, 2, 3, 4 or >5 turns of a grinder

“Not sure because it is added during food preparation” and “Not sure/I don’t pay attention” were included in frequency and portion size questions to reduce the likelihood of guessing.

Information gathered from the HSQ included total number of herbs/spices consumed and frequency and portion size used per month. Portion sizes were converted to gram weights using USDA Food Data Central [18], which lists gram weight per teaspoon for herbs and spices. Testing was performed by the investigator to determine the approximate average percentage of a small-hole-container shake of an herb/spice as 6% of a teaspoon. Thus, each small-hole-container shake was calculated as 0.06* grams/teaspoon of a particular herb/spice and each medium- and large-hole-container shake was calculated as 0.08* grams/teaspoon and 0.12* grams/teaspoon, respectively. For comparisons to food records and 24-hr recall herb and spice frequency, as well as portion measures, per-month consumption of herbs and spices from the HSQ was divided by 30 to estimate daily intakes. Responses of “Not Sure” for frequency and portion consumed were left blank in the data files. No data were collected regarding the use of herbal infusions.

### 2.4. Relative Validity Testing

Relative validity was first evaluated by comparing herb and spice measurements collected by the HSQ to those collected by two comparator methods: 7-day food records and three 24-h recalls. Two separate groups of participants were used for validity testing, with one completing the HSQ and food records and the other completing the HSQ and food recalls.

Food records were completed using eaTracker^®^, Dietitians of Canada’s (Toronto, Canada) online nutrition and physical activity tracking program. The eaTracker food database contained all herbs and spices included in the HSQ except for annatto and turmeric. Participants were instructed to record herb and spice intake in addition to food and beverage for seven consecutive days. Items consumed but not found in eaTracker were recorded on a separate digital log. Research staff retrieved participants’ food records and nutrient analyses generated by eaTracker’s database, the Canadian Nutrient File version 2015 [19]. Food records were examined for herb and spice use and frequency and amounts were recorded by research staff on study datasheets. Teaspoon and shaker portions were converted to gram amounts as detailed above under “Herb and Spice Questionnaire.” Herb and spice intakes for the 7 days were considered to be representative of a week’s intake and measures were multiplied by 4 to estimate monthly intake. Note that the eaTracker program used in this study was discontinued on 15 December 2019.

24-h dietary recalls were completed using the Automated Self-Administered 24-h Dietary Assessment Tool (ASA24^®^, version 2018) developed by the National Cancer Institute, Bethesda, MD, USA [20]. Participants completed three scheduled ASA24s administered by telephone by a research staff member. The same staff member completed all recalls. For each participant, recalls collected data for two non-consecutive weekdays and one weekend day within the same week. Participants were asked during each recall if they consumed herbs or spices and what amounts were used during food preparation or added at meal consumption. Recalls were analyzed by ASA24 using the Food and Nutrient Database for Dietary Studies (FNDDS) 2011–12 and 2013–2014 [21]. Teaspoon and shaker portions were converted to gram amounts as detailed above under “Herb and Spice Questionnaire.” Spice and herb intakes for the three days were considered to be representative of a week’s intake and measures were multiplied by 4 to estimate the monthly intake.

Relative validity of the HSQ was also evaluated by determining the correlation of herb and spice consumption with diet quality. Evidence supporting a positive correlation between herb and spice intake and diet quality exists in the findings that spice consumers tend to score higher on the Dietary Approaches to Stop Hypertension (DASH) index [22], and that a more anti-inflammatory diet (which includes herbs and spices) is positively correlated with higher scores on multiple diet quality indices including the Healthy Eating Index-2010 (HEI-2010), Alternative Healthy Eating Index and the DASH index [23].

In the current study, diet quality was assessed by the HEI-2015 score calculated from participants’ answers on the National Cancer Institute’s online Diet History Questionnaire version III (DHQ III, National Institutes of Health, Applied Research Program, National Cancer Institute, Bethesda, MD, USA, 2018) examining the past month with portion size [24]. The HEI-2015 assesses diet quality according to adherence to the US Dietary Guidelines, with scores ranging from 0 (no adherence) to 100 (optimal adherence). Participants in both comparator method groups (food records; 24-h recalls) completed the DHQ III. Output data from the DHQ III included HEI-2015 total and individual component scores.

### 2.5. Data Analysis

Consumption of total and individual herbs and spices from the HSQ was calculated as frequency and gram amount in times per month and day. Herb and spice intakes from food records and 24-h recalls were averaged across seven days and calculated on a per-month and per-day basis for both total and individual herb/spice intakes. Herb and spice data were skewed; therefore, the non-normally distributed were transformed prior to analysis. Wilcoxon signed-rank tests were used to test for significant differences between means on Bland–Altman plots [25]. Differences between the HSQ and each comparator method were also examined using analysis of variance (ANOVA). Use or non-use of individual herbs and spices was compared between the HSQ and comparator methods using logistic regression with likelihood ratio tests. Results were considered to be statistically significant at *p* < 0.05.

## 3. Results

### 3.1. Participants

One hundred twenty-six individuals responded to the study advertisement. Twelve of these were not eligible due to adherence to a restricted diet and 52 did not complete all study activities. The final number of participants with complete data was 62 (33 completed food records as the comparator method and 29 completed 24-h recalls as the comparator method).

### 3.2. Herb and Spice Use Across Methods

All but one participant reporting consuming at least one herb/spice on any of the HSQ, food records or 24-h recalls (range 0–20), and the most common number of herbs/spices reported was 13 (reported by 8 participants on the HSQ). All herbs and spices listed on the HSQ except dill were reported as consumed by at least one participant. Of 500 affirmative responses of herb and spice use on the HSQ, 97 (19.4%) did not specify the portion size and 2 did not specify frequency (response “Not Sure”).

### 3.3. Relative Validity

The HSQ ranked the same three herbs and spices as the most commonly consumed as did the food records and 24-h recalls: black pepper as first, and cinnamon and garlic powder as second or third, Table 1 and Table 2. The percentage of participants reporting use of black pepper and garlic powder on the HSQ vs. 24-h recalls was not significantly different. However, a significantly lower percentage of participants reported use of cinnamon on the 24-h recalls vs. HSQ (*p* < 0.01). The percentage of participants reporting use of these three spices on food records was significantly less than the percentage reporting use on the HSQ (*p* < 0.001).

Analyses performed using ANOVA showed significantly higher measures of number of herbs and spices consumed, total consumption per month and day and frequency of consumption recorded by the HSQ vs. each of the comparator methods, Table 3 and Table 4. While ANOVA showed no significant difference in portion size between the HSQ and food records (*p* > 0.05), analysis using the Wilcoxon signed-rank test (see below) revealed a significant lack of agreement between methods.

**Figure 1:**
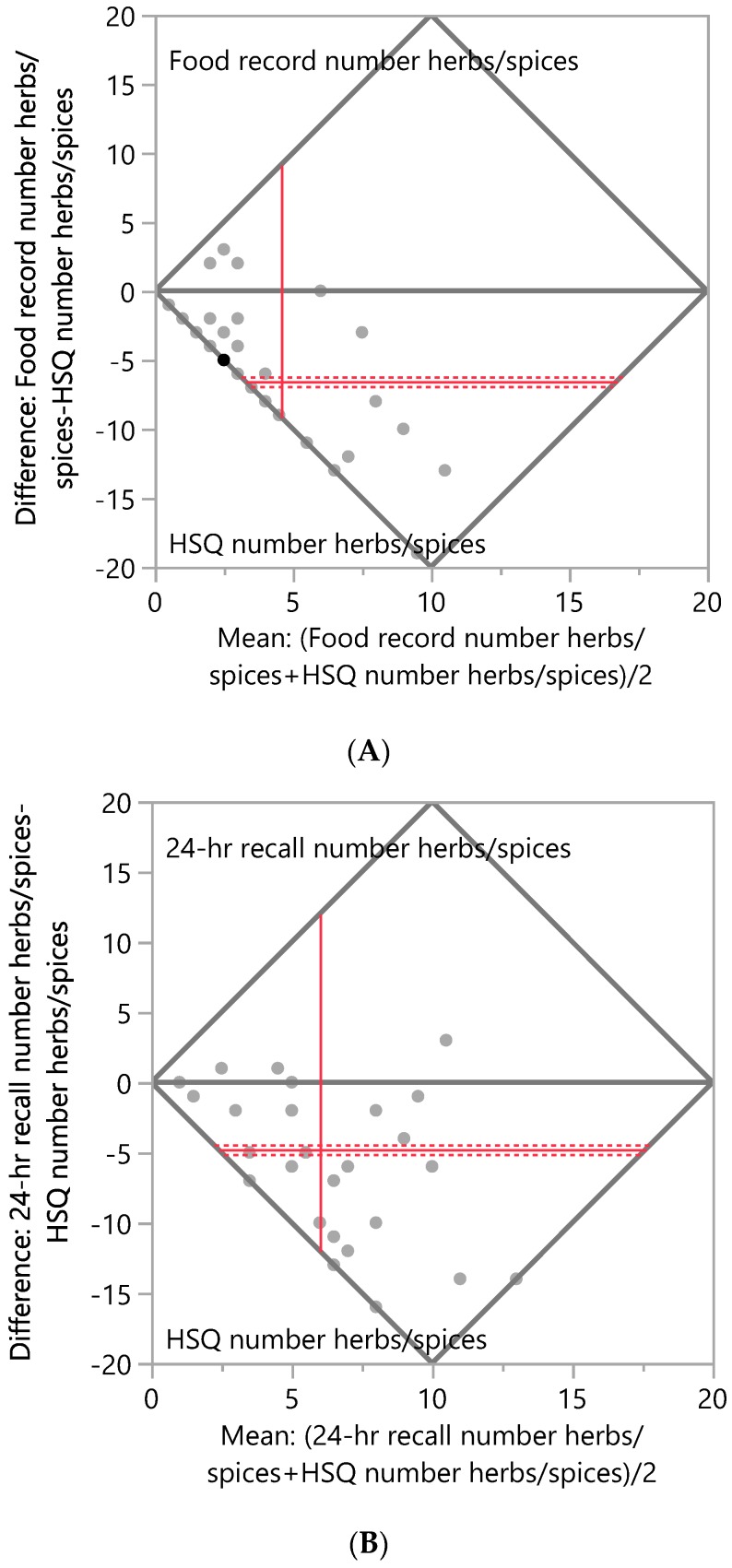
Bland–Altman Plots of the number of herbs and spices consumed per month. (**A**) Difference in number of herbs and spices consumed between the Herb/Spice Questionnaire (HSQ) and food records. N = 33 respondents; mean difference between methods = −6.5, Wilcoxon signed rank test, *p* < 0.0001. (**B**) Difference in number of herbs and spices consumed between the HSQ and 24-h recalls. N = 29 respondents; mean difference between methods = −4.7, Wilcoxon signed rank test, *p* < 0.0001. The mean difference is shown as the horizontal solid red line, with the 95% confidence interval above and below shown as red dotted lines. The mean of pairs is shown by the vertical red line.

**Figure 2:**
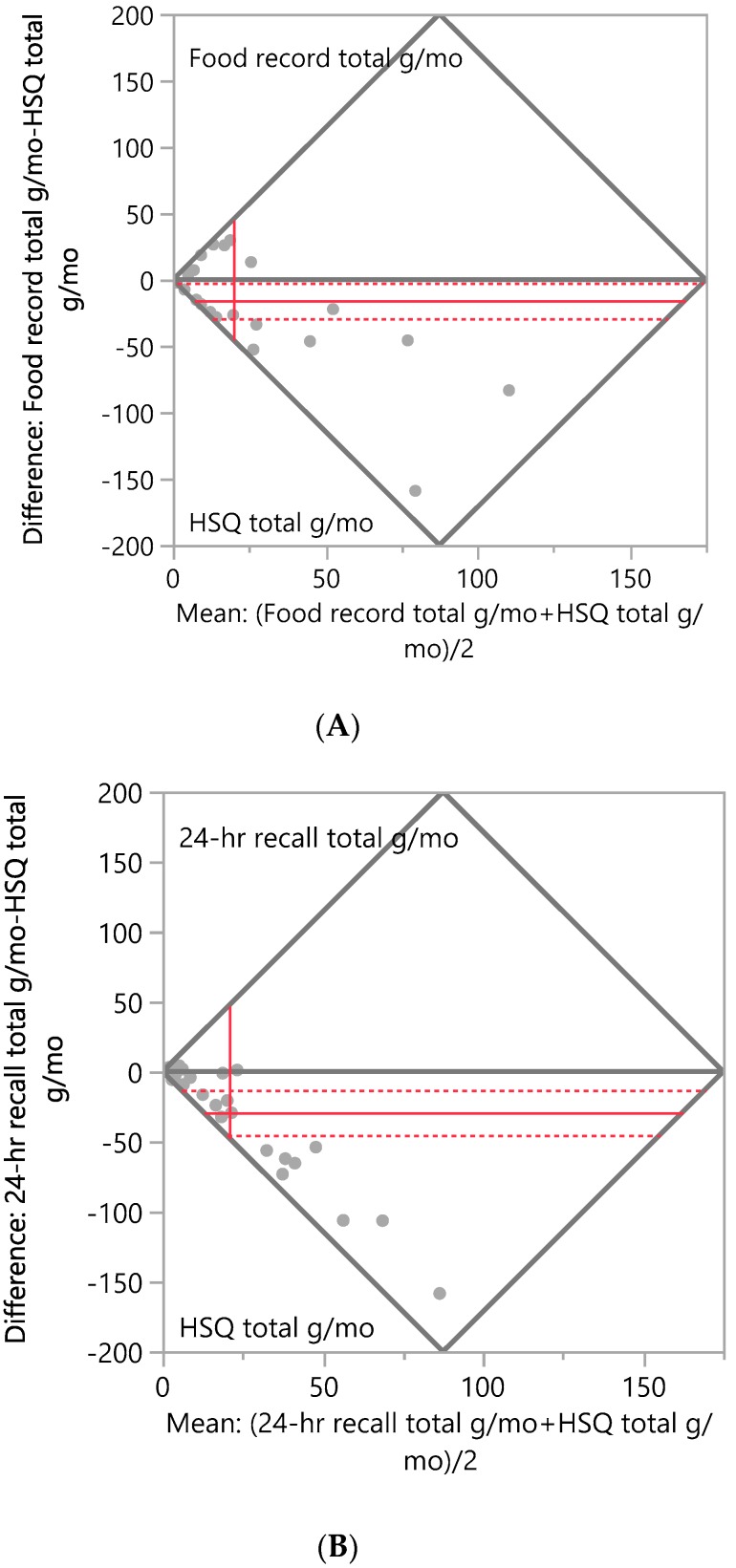
Bland–Altman Plots of the total grams of herbs and spices consumed per month. (**A**) Difference in total g/mo of herbs and spices consumed between the Herb/Spice Questionnaire (HSQ) and food records. N = 33 respondents; mean difference between methods = −15.8, Wilcoxon signed rank test, *p* < 0.05. (**B**) Difference in total g/mo of herbs and spices consumed between the HSQ and 24-h recalls. N = 29 respondents; mean difference between methods = −29.2, Wilcoxon signed rank test, *p* < 0.001. The mean difference is shown as the horizontal solid red line, with the 95% confidence interval above and below shown as red dotted lines. The mean of pairs is shown by the vertical red line.

Bland–Altman plots, Figure 1A,B, illustrate that the number of herbs and spices reported was significantly higher according to the HSQ vs. both comparator methods: mean difference between 24-h recalls and HSQ = −4.7, *p* < 0.0001; mean difference between food records and HSQ = −6.5, *p* < 0.0001. The total amount of herbs and spices consumed in g/mo was significantly different according to the HSQ vs. the comparator methods: HSQ vs. 24-h recalls mean difference = −29.2, *p* < 0.001; HSQ vs. food records mean difference = −15.8, *p* < 0.05, Figure 2A,B. When gram amounts consumed were calculated on a per-day basis, significant differences between the HSQ and the comparator methods were also seen: HSQ vs. 24-h recalls g/d mean difference = −1.0, *p* < 0.001; HSQ vs. food records mean difference = −0.5, *p* < 0.05. Frequency of herb and spice consumption per month was significantly less for the HSQ compared to the estimated frequency for the comparator methods: mean difference between 24-h recalls and HSQ = 0.5, *p* < 0.0001; mean difference between FR and HSQ = 6.0, *p* < 0.0001. Portion size measured by the HSQ was significantly larger than that measured by 24-h recalls (mean difference = −0.6, *p* < 0.0001) and food records (mean difference = −0.5, *p* < 0.05).

**Figure 3:**
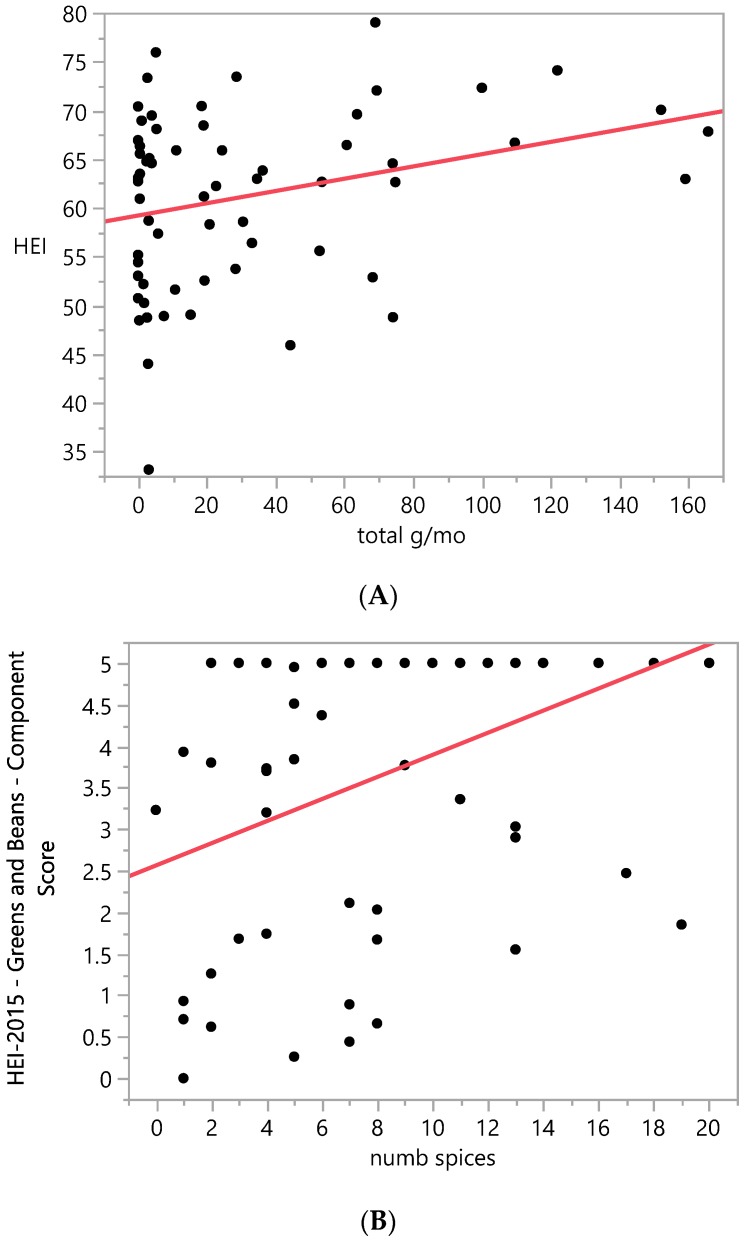
Correlations between Herb and Spice Questionnaire measurements and Healthy Eating Index-2015 score. (**A**) Correlation between total g/mo consumption of herbs/spices as recorded by the Herb and Spice Questionnaire (HSQ) and Healthy Eating Index-2015 (HEI) total score. N = 62. Pearson correlation coefficient = 0.3, *p* < 0.05. (**B**) Correlation between HEI Greens and Beans component score and number of herbs/spices consumed per month as recorded by the HSQ. Pearson correlation coefficient = 0.4, *p* < 0.0001. N = 62.

Correlational analysis showed a modest, significant positive correlation between total HEI and total g/mo consumption of herbs and spices recorded by the HSQ, with Pearson correlation coefficient = 0.3, *p* = 0.02, Figure 3A. The HEI component scores for greens + beans and total vegetables were also significantly correlated with HSQ total g/mo intake, both with Pearson correlation coefficient = 0.3, *p* = 0.02. HSQ number of spices consumed per month was weakly correlated with the HEI component score for total vegetables (Pearson correlation coefficient = 0.2, *p* < 0.0001) and modestly correlated with HEI component score for greens + beans: Pearson correlation coefficient = 0.4, *p* < 0.0001, Figure 3B. Total HEI and number of spices consumed per month were not significantly correlated.

## 4. Discussion

This initial validation study of a newly created online questionnaire generated findings that are valuable for moving forward in the development of an accurate, reliable herb and spice dietary assessment tool. The HSQ proved feasible to administer using an online platform. Respondents spent an average of 5 min completing the HSQ and individual herb and spice measurements were collected for analysis as expected. The HSQ performed well in detecting use of commonly consumed herbs and spices, but quantitative measurements of intake were generally significantly higher for the HSQ vs. the comparator methods. In a second test of relative validity, herb and spice measures collected by the HSQ showed a fair correlation with HEI score and select component scores.

The HSQ matched 24-h recalls and food records in identifying the same three spices, black pepper, garlic powder and cinnamon, as the most commonly consumed herbs and spices among respondents. These results are similar to those reported elsewhere [14,16,22]. In their study of Americans’ knowledge, perceptions and use of spices, Isbill et al. [16] found that 57%, 41% and 15% of respondents used black pepper, garlic and cinnamon, respectively, daily. In an Iranian population, Hashemian et al. [22] reported black pepper and cinnamon use by 70% and 30% of participants, respectively.

Two reasons for the lack of agreement between the HSQ and comparator methods can be proposed including the difference in level of inquiry regarding herb and spice intake. The HSQ required responses to questions about consumption of multiple individual herbs and spices while the comparator methods required respondents to recall herb and spice use with minimal prompting. Despite receiving instructions that included a list of herbs and spices in the eaTracker food database and directions to record all herb and spice use, participants rarely reported herbs or spices on food records. Participants completing telephone-administered 24-hr recalls vs. food records were more likely to report herb and spice use, but reported intakes were still significantly lower compared to those recorded by the HSQ.

Another proposed reason for the discrepancy in estimates of herb and spice intake between the HSQ and the comparator methods is the difference in time period of dietary assessment: 30 days for the HSQ vs. 3 days and 7 days for the 24-h recalls and food records, respectively. The decision not to use 30-d food records and thrice-weekly 24-h recalls over four weeks was based on the investigator’s concerns of excessive participant burden and diminishing accuracy of dietary assessment data over time. Estimations of habitual herb and spice intakes from 7-d food records and three 24-h recalls may have missed consumption occasions that occur sporadically. Indeed, Carlsen et al. found good correlations in spice and herb intakes between their herb/spice questionnaire and 28-d herb/spice records [14].

The present study suggests that the HSQ holds potential for addressing the need for a dietary assessment tool specific to habitual herb and spice consumption. The findings contribute to the field by demonstrating the feasibility of collecting detailed herb/spice intake data online. The results, showing that all but one of the 62 respondents reported using at least one herb/spice on the HSQ and that 80% of respondents provided specific amount and frequency information, support the usability of the HSQ format and administration method. All participants are shown to have completed the questionnaire once started; therefore, the length appears to be acceptable to respondents. Further, the list of herbs and spices included in the HSQ appeared to be complete based on the fact that only two participants used the add-in option. The finding that dill was not used by any participant suggests that this herb could be removed from the questionnaire. The current findings are also important in showing the need for a robust comparator method of measuring herb/spice intake and careful participant selection. The commonly used dietary assessment methods of 24-h recalls and food records did not capture herb/spice intakes at nearly the same level as the HSQ. A contributor to the poor rate of herb/spice reporting on the comparator methods may be a possible marginal motivation on the part of participants to provide detailed, complete dietary information across multiple individual days. The study completion rate of ~55% of initial enrollees and the considerable attention to participant follow-up required by research staff indicate that stricter screening criteria should be employed in future studies of the HSQ. For example, personal interviews with potential participants should clearly address the level of time and commitment needed and, optimally, include an incentive for each participant. The current study offered a random award drawing for participants, which may not have been sufficient to promote best efforts in completing the relatively demanding food records and 24-h recalls.

Strengths of the present study are the use of multiple comparator methods for relative validity testing and the fully online format of the HSQ. Limitations include the small sample size, different time periods of assessing herb and spice intake across methods, risk of bias in dietary self-report and the absence of demographic data for respondents. The lack of information on participant sex, age and other personal characteristics restricts the ability to assess internal or external validity of the HSQ. Without identifying attributes of respondents, it is unknown how the HSQ would perform in other populations. Future validation testing ideally would target a specific and clearly defined population, would utilize a comparator method encompassing the same 30-d period as the HSQ and would include probes for herb and spice use for each eating occasion throughout the assessment period. Also, the population tested ought to be expanded in number to improve power and enable stratification by factors that may impact results.

Lastly, the importance of continuing to advance the quality and breadth of self-administered/self-reported dietary assessment tools is seen in the challenges of developing objective dietary biomarkers. Identifying and refining objective measures of dietary exposure are goals in nutrition research and impressive progress has been made in designing methodologies for assessing intakes of energy [26], sugar, protein, sodium [27] and potassium [28]. However, many biomarkers of food intake are non-specific and/or significantly affected by factors such as host genome and gut microbiota [3]. The transformation of compounds in herbs and spices into a large number of metabolites by gut microbiota poses a particular challenge to identifying biomarkers of herb and spice intake [29,30,31]. Difficulty in detecting herb and spice metabolites in biofluids is compounded by the pattern of their typical consumption, which is sporadic and in small quantities [3]. Thus, the concurrent development of new dietary assessment tools, such as the HSQ, and biomarkers is critical to the progress of nutrition research.

## 5. Conclusions

In conclusion, this validation study showed good performance of a new online herb and spice questionnaire in detecting use of specific commonly consumed herbs and spices. Measures of herb and spice use collected by the HSQ also correlated in an expected manner with diet quality. However, the HSQ recorded significantly higher measures of intake than the comparators, which indicates that further validating testing is needed before the HSQ is implemented in research studies. The findings suggest that the format, length and question content (list of herbs and spices; amount and frequency options) of the HSQ are appropriate but that careful selection of motivated respondents is needed for robust validity testing. A future goal is to incorporate an herb and spice questionnaire into an existing validated online dietary assessment method.

## Figures and Tables

**Table 1:** Comparison between the herb/spice questionnaire and the food records of reported use of the most commonly consumed herbs and spices.

	Percentage of Participants Reporting Use by Dietary Assessment Method, N (%)	
Spice	Herb/Spice Questionnaire	Food Records
Black Pepper	26 (79%) *	11 (33%)
Cinnamon	21 (64%) *	6 (18%)
Garlic	21 (64%) *	7 (21%)

N = 33 respondents. Likelihood ratio test between methods within rows, * *p* < 0.001.

**Table 2:** Comparison between the herb/spice questionnaire and the 24-h recalls of reported use of the most commonly consumed herbs and spices.

	Percentage of Participants Reporting Use by Dietary Assessment Method, N (%)	
Spice	Herb/Spice Questionnaire	24-h Recalls
Black Pepper	25 (86%)	22 (76%)
Cinnamon	21 (72%) *	9 (31%)
Garlic	16 (55%)	17 (59%)

N = 29 respondents. Likelihood ratio test between methods within rows, * *p* < 0.01.

**Table 3:** Mean and median herb and spice intakes collected by the herb/spice questionnaire and food records.

Measurement		Herb/Spice Questionnaire	Food Records	
	Mean (SD)	Median (IQR)	Mean (SD)	Median (IQR)
Number of herbs & spices/mo	7.8 (4.7) ^a^***	7 (8)	1.3 (1.9) ^b^	0 (2)
Consumption, total g/mo	26.9 (41.4) ^a^*	4.0 (37.4)	11.5 (17.7) ^b^	0.0 (20.1)
Consumption, total g/d	0.9 (1.4) ^a^*	0.1 (1.2)	0.4 (0.6) ^b^	0.00 (0.7)
Frequency of consumption/mo	2.8 (4.2) ^a^***	2 (2.5)	7.9 (4.5) ^b^	8 (8)
Portion size (g) per use	0.8 (0.9)	0.5 (0.8)	0.6 (0.6)	0.5 (0.9)

N = 33. ^a,b^ Mean values with different superscript letters are significantly different by analysis of variance (ANOVA); *** *p* < 0.0001, * *p* < 0.05. SD, standard deviation; IQR, interquartile range.

**Table 4:** Mean and median herb and spice intakes collected by the herb/spice questionnaire and 24-h recalls.

Measurement	Herb/Spice Questionnaire		24-h Recalls	
	Mean (SD)	Median (IQR)	Mean (SD)	Median (IQR)
Number of herbs & spices/mo	8.4 (5.0) ^a^***	9 (6)	3.6 (2.9) ^b^	3 (4)
Consumption, total g/mo	36.5 (42.9) ^a^**	20.8 (66.8)	6.4 (6.4) ^b^	4.2 (6.4)
Consumption, total g/d	1.2 (1.4) ^a^**	0.1 (0.2)	0.2 (0.2) ^b^	0.1 (0.2)
Frequency of consumption/mo	5.9 (14.0) ^a^**	2 (3.5)	5.9 (2.7)	4 (4)
Portion size (g) per use	0.7 (0.8) ^a^***	0.5 (0.8)	0.1 (0.2) ^b^	0.6 (0.0)

N = 29. Within rows, ^a,b^ values with different superscript letters are significantly different by ANOVA; *** *p* < 0.0001, ** *p* < 0.001. SD, standard deviation; IQR, interquartile range.
